# Pre and Post-Operative Alterations of the Gastrointestinal Microbiome Following Bariatric Surgery

**DOI:** 10.7759/cureus.13057

**Published:** 2021-02-01

**Authors:** James M Santos, Meby S Mathew, Nilam Shah, Renzo Pajuelo-Vasquez, Anuja Mahesh Mistry, Stacey E Heindl

**Affiliations:** 1 Medicine, California Institute of Behavioral Neurosciences & Psychology, Fairfield, USA; 2 Pharmacy Practice, California Institute of Behavioral Neurosciences & Psychology, Fairfield, USA; 3 Pharmacy Practice, Nirmala College of Pharmacy, Muvattupuzha, IND; 4 Neurology, California Institute of Behavioral Neurosciences & Psychology, Fairfield, USA; 5 Internal Medicine, Smt N.H.L Municipal Medical College, Ahmedabad, IND; 6 Medicine, Avalon University School of Medicine, Willemstad, CUW

**Keywords:** bariatric surgery complications, gastrointestinal microbiome, upper gastrointestinal surgery, gut microbiome, gut microbiome (metagenomics) and metabolomics aspects of diabetes, endoscopic management of obesity, laparoscopic roux-en-y gastric bypass, adjustable gastric band complications, gastric bypass surgery, bariatric surgery/therapeutic use

## Abstract

Obesity in the United States is increasing at a startling rate, with more individuals turning towards bariatric surgery as treatment. A noteworthy aspect of obesity pathology is its interplay with the gastrointestinal microbiome. The gastrointestinal microbiome comprising trillions of microorganisms affects the dynamics of digestion, energy expenditure, and neurologic mechanisms that affect dietary preference. This literature review used PubMed to search for articles about obesity, gastrointestinal microbiome, and bariatric surgery. The researchers used Medical Subject Heading keywords, and then the relevant literature was selected and filtered using inclusion and exclusion criteria. This study aims to review the temporal relationship of gastrointestinal microbiome changes after bariatric surgery in association with the success and failure of treatment along with the factors that may have altered the gastrointestinal microbiome other than the anatomical aspect of bariatric surgery.

## Introduction and background

Obesity in the United States has increased from 30.5% to 42.4% in 20 years and is growing upward. Among the different categories of obesity, an alarming rate is seen in severe obesity, which has increased from 4.7% to 9.2% [1]. Obesity is known as a condition brought about by an overabundance of body fat. However, new studies have described obesity as a dysfunction of the constant shifting of homeostatic control of energy balance towards a positive energy balance [2]. Thus, proving that obesity has a multifactorial and complex etiology.

The gut microbiota comprises more than 10-100 trillion microorganisms, which contains the most significant number of microorganisms seen in the human body. Recent studies have identified that there are alterations at the phylum level in obese individuals [3]. Proposed mechanisms for intestinal microbiota-induced obesity are increased energy harvesting by the microbiota, changes in metabolic pathways, and induction of low-grade inflammation [4]. Apart from obesity, the intestinal microbiome affects the outcome of gastrointestinal organ development, immune system maturation, bone homeostasis, and physiologic signaling, among other developmental processes, as well as pathologic processes such as inflammatory pathology, cardiovascular diseases, and obesity [5].

Clinicians may manage obese patients through nutrition, physical activity, behavior therapy, pharmacotherapy, and bariatric surgery [6]. The first clinical recommendation for obese patients is to increase physical activity in combination with diet and behavioral modifications. However, patients with a body mass index (BMI) of 30 kg/m^2^ or greater and 27 kg/m^2^ or greater who have comorbidities and have unsuccessfully tried diet and physical activity should be prescribed medications for weight loss. Patients with further increased BMI of 40 kg/m^2^ or greater and 35 kg/m^2 ^who have comorbidities can undergo bariatric surgery [7].

Bariatric procedures in the United States in the last seven years have increased by 60%, with an estimated 252,000 procedures done in a year [8]. Sleeve gastrectomy (SG), Roux-en-Y gastric bypass (RYGB), and laparoscopic adjustable gastric band (AGB) make up most of the procedures [9]. The effects of these procedures are due to caloric restriction seen in manipulating the stomach into a smaller pouch or the malabsorption observed due to the anatomical change. However, some of the unforeseen effects of bariatric surgery ranging from a sensation of reduced hunger and change of food preference suggests that neither of the previously attributed mechanisms can explain this association, suggesting that there are possibilities of alteration of communication between the gastrointestinal tract and neurocircuits in energy homeostasis involved in the “gut-brain axis” [2,4,10].

Bariatric surgery not only helps in achieving persistent weight loss but also aids in the improvement of diabetes, dyslipidemia, and hypertension in obese patients. However, some cases led to failed maintenance of weight loss after bariatric surgery in the long term. These cases are classified as failed bariatric surgery when there is <25% excess weight loss at more than five years from the procedure [11].

There are five identifiable possible causes of weight regain following bariatric surgery: endocrine and metabolic alterations, anatomic surgical failure, nutritional non-adherence, psychological factors, and physical inactivity [12]. Bariatric procedures are known to alter the intestinal microbiome. Some intestinal bacteria promote more efficient absorption of nutrients, facilitate central nervous system signaling that may influence energy balance, influence bile acid metabolism, and gut hormone secretion, affecting energy balance [13].

Current studies on animals have demonstrated that manipulating intestinal microbiota through fecal transfusion from post-bariatric surgery patients can change the host behavior by changing food preference. Human studies have shown alteration in the microbiome and its effects on metabolism [4,9]. Due to the diverse microbiome and external factors that affect the intestinal microbiota in humans, studies have not shown the same substantial evidence of causality to obesity such as in murine studies [2,10].

This review aims to understand the intestinal microbiome changes seen after bariatric surgery and focus on the temporal relation of the quantitative and qualitative findings seen post-surgery.

## Review

Methods

This integrative review of literature searched the PubMed database using MeSH keywords and general search terms, including “gut microbiome,” “bariatric surgery,” and “gastrointestinal microbiome,” which were used both individually and in combination.

The original articles used in the study were published between 2015 and 2020. All of the articles were published in English. Part of the preliminary screening was to look for studies that were readily available online. We evaluated observational studies and clinical trials that assessed the gut microbiota of obese adults before and after bariatric surgery.

This list generated several hundred pieces of literature, from which we manually assessed and omitted some articles, which were case reports, case series, systematic reviews, and meta-analyses. Table 1 contains a quantitative list of studies that we encountered during the research process.

**Table 1:** Keywords and search terms used during the literature search.

MeSH keywords and general search terms	Number of articles
Bariatric surgery	26,416
Gastrointestinal microbiome	17,478
Gastrointestinal microbiota AND bariatric surgery	109
Microbiome AND gastric bypass	60
Gut microbiome AND gastric bypass	46
Total articles used after inclusion and exclusion criteria	150

Results

We found 12,120 articles based on individual and combined MeSH search terms. We then filtered the studies using our inclusion and exclusion criteria. We included human research studies published between 2015 and 2020. This study focuses on adult obesity and bariatric surgery. We limited the search to articles that were readily accessible to the general public. We excluded studies that were not in English as well as those that focused on the pediatric population. We were able to narrow the articles down to 150 articles.

The articles were then manually reviewed, and the researchers removed all duplicates and case reports, case series, systematic reviews, and meta-analyses. After further review, we saw that 14 studies fit our inclusion and exclusion criteria and aligned with this review’s focus. Table 2 contains a summary of the articles included in this study.

**Table 2:** Summary of the included articles. RYGB = Roux-en-Y gastric bypass; SG = Sleeve gastrectomy; MWL = medical weight loss; AGB = adjustable gastric banding; LAGB = laparoscopic adjustable gastric banding; VGB = vertical banded gastroplasty; GB = gastric banding; LSG = laparoscopic sleeve gastrectomy; MT = medical dietary treatment

Authors and year of publication	Intervention	Number of patients	Results	Conclusion
Steinert et al. [14]. 2020	RYGB	25	There was a noticeable difference in the gut microbiota between obese patients before surgery versus healthy controls and after surgery, with a significant unidirectional shift. As for fungal microbiota, there were no significant differences between groups, but there were individualized changes in the fungal microbiota.	RYGB surgery may have caused a shift in microbial microbiota because of the change in gastrointestinal conditions to which bacteria are susceptible. On the other hand, fungal microbiota is aerobic and less sensitive to increase in oxygen availability after surgery, thus having no significant differences between groups. Another factor that could’ve affected the microbial and fungal microbiota is the eating habits of the patients. Daily diet has a strong influence on gut microbiota.
Pajecki et al. [15]. 2019	RYGB	9	In 15 months, patients who underwent RYGB had a mean of 55.9% excess weight loss. The majority of the microbiota showed a significant reduction in *Proteobacteria *and no changes in *Firmiticus *and *Bacteroidetes*. In two individuals, microbiota had an increase in *Firmiticus *and a decrease in *Bacteroidetes*. These patients had less weight loss compared to the rest of the group.	Gastric bypass surgery changes the microbiota of super-obese patients. However, different bacteria contribute to the process of varying weight loss groups.
Assal et al. [16]. 2020	RYGB	25	After RYGB, the gut microbial richness increased, and the *Firmiticus*/*Bacteroidetes *ratio decreased, regardless of type 2 diabetes remission. Richness level correlated with dietary habits in pre- and post-RYGB, showing positive and inverse correlation with fiber and lipid intakes.	Gut microbiota richness increased after RYGB and altered its bacteria genus profile, but these changes did not correlate with type 2 diabetes mellitus remission. Changes in the particular gut microbiota, which is potentially induced by dietary habit changes, may be crucial in predicting type 2 diabetes mellitus after RYGB.
Shen et al. [17]. 2019	RYGB and SG	26	Bariatric surgery brought rapid gut microbiome diversity and composition changes with a steady increase until six months, which then decreased by 12 months. However, the decrease at 12 months was still higher than pre-surgery. There was an abundance of *Verrucomicrobia *and *Proteobacteria *after surgery.	The gastrointestinal microbiome acts synergistically to modulate circulating biomarkers, thus, decreasing circulating biomarkers of inflammation, increasing bile acids and choline metabolism products. Microbiome composition, diversity, and function tended to regress to pre-surgery levels one year after surgery.
Gutiérrez-Repiso et al. [8]. 2019	RYGB	24	Patients were divided into three groups: success weight loss, primary failure weight loss, and weight regain. The success group noted the highest abundance of *Bacteroidetes*, *Firmiticus*, *Proteobacteria*, and *Actinobacteria *versus the weight regain group, which had the lowest quantity. Also, patients from the success group had a more diverse core microbiome. Among the genera, *Sarcina *abundance was closely correlated to BMI and cholesterol metabolism post-surgery.	The gastrointestinal microbiome has a pivotal role in maintaining BMI and cholesterol metabolism, possibly through bile acids seen after surgery, suggesting that the microbiome changes mediate the success rate seen after surgery on weight loss.
Fouladi et al. [18]. 2019	RYGB	18	There was an increased abundance of *Actinobacteria *and *Firmiticus *in the successful weight loss and poor weight loss groups. There was significant weight gain and a higher quantity of *Barnesiella *among the poor weight loss group in the antibiotic-treated mice given the gastrointestinal microbiota from RYGB patients.	While there was no substantial difference in the gut microbiota composition among successful weight loss and poor weight loss groups, there were still evident differences in functionality. This suggests that some taxa may contribute to weight gain after RYGB, which were prominent in the poor weight loss group.
Aron-Wisnewsky et al. [19]. 2019	ABG and RYGB	61	Low microbial gene richness was seen in most patients, especially those with increased trunk-fat mass and comorbidities. The common metagenomic species were coupled with adverse body composition and metabolic phenotypes. One year after surgery, there was an increase in microbial gene richness. However, most RYGB patients remained with low microbial gene richness, despite having better metabolic improvement than AGB patients.	Severe obesity is associated with a low microbial gene richness and low metagenomic species linked with visceral adiposity, adipocyte hypertrophy, and metabolic and inflammatory deterioration. Bariatric surgery improves microbial gene richness, but it restores to being low, despite notable metabolic improvement and weight reduction in most patients.
Ilhan et al. [20]. 2017	RYGB and LAGB	63	RYGB group had a significantly different microbiome than the average weight, pre-bariatric surgery morbidly obese, and LAGB groups. There was more diversity in the RYGB group than the LAGB, including *Escherichia*, *Veillonella*, and *Streptococcus*. Amino acid and carbohydrate fermentation products were prevalent in the RYGB group.	Compared to the other groups, the difference seen in the post-RYGB group is attributed to various environmental conditions caused by the profoundly transformed gastrointestinal anatomy. It provides evidence that gastrointestinal anatomy changes lead to a different microbiome diversity of amino acid and carbohydrate fermentation.
Palleja et al. [21]. 2016	RYGB	13	There was an increase in gut microbial diversity and altered microbial composition seen in the first three months after RYGB and maintained during the following nine months, albeit the rate of improvement was noticeably lower. Most of the microbial species increased, especially *Escherichia*, *Klebsiella*, *Veillonella*, *Streptococcus*, *Alistipes*, and *Akkermansia*. *Faecalibacterium* was the only species that decreased in abundance.	The changes in the gut microbiota after RYGB are in parallel with weight loss and metabolic improvements. The study noted functional changes observed after surgery, such as using multiple energy sources using transporters and phosphotransferase systems, were reported. The researchers observed aerobic respiration, the shift from protein degradation to putrefaction, and amino acids and fatty acids as energy sources.
Tremaroli et al. [22]. 2015	RYGB and VGB	21	The researchers colonized germ-free mice with stool from patients who underwent RYGB or VBG. The results showed that the microbiota promoted reduced fat mass deposition in the inoculated mice. There was also a decreased utilization of carbohydrates as fuel.	Both RYGB and VBG had long-term changes in the gut microbiome independent of BMI and regulated metabolism and reduced fat mass deposition.
Paganelli et al. [23]. 2019	RYGB and SG	45	After RYGB and SG, there was a significant decrease in the BMI of all patients. Crash diet caused microbiota diversity to decline, but it gradually returned at six months after surgery. There was a significant increase in *Streptococcaceae *and *Enterobacteriaceae* families and decreased *Bifidobacteriaceae *post-surgery, which persisted until six months. Other clinical parameters, such as serum level of Vitamin D and B6, cholesterol, bilirubin, HbA1c, iron, ferritin, and folate, improved six months after surgery.	Crash dieting showed a temporary change in the microbiota. Bariatric surgery, whether RYGB or SG, had a permanent change in microbial diversity, which may have contributed to the significant decrease in weight and possible improvement in some clinical parameters.
Dao et al. [24]. 2019	RYGB and GB	65	*Akkermansia *was significantly lower in severely obese patients than in moderately obese patients but had no association with glucose homeostasis. After surgery, there was an upward trend in the abundance of *Akkermansia*. There was no association between the quantity of *Akkermansia *with glucose homeostasis both before and after surgery.	Even though *Akkermansia *abundance is lower in severe obesity than RYGB, there is no correlation with metabolic improvement. There may be a certain threshold to be attained for noticeable post-surgical improvement towards a healthier clinical profile.
Sanmiguel et al. [25]. 2017	LSG	8	LSG led to significant reductions in BMI, food intake, and hedonic eating. *Biliophila *and *Faecalibacterium *were associated with weight loss. *Enterococcus *was related to lower appetite, and *Akkermansia *was related to reduced hedonic eating post-surgery.	The post-operative shift in gut microbial composition suggests that bariatric surgery affects the gut microbiota-brain interactions axis, possibly associated with the changes in weight, appetite, and hedonic eating parameters.
Medina et al. [26]. 2017	MT, RYGB, and SG	19	There were no significant changes in the gut microbiota of the medically treated group. Both RYGB and SG groups had substantial alterations in their gut microbiota. *Proteobacteria *increased after six months in both RYGB and SG, while *Bacteroidetes* increased in RYGB but decreased SG. These changes correlated positively with specific anthropometric or metabolic parameters.	The gastrointestinal tract’s physiologic rearrangement probably causes significant changes in the gut microbiota of both surgical groups. The RYGB and SG groups altered the gut microbiota differently, and these adjustments may contribute to weight and metabolic improvement.

Discussion

Obesity and Gastrointestinal Microbiome

In recent years, the pathogenesis of obesity has changed from being viewed as a storage disease to now a brain-centered problem. The brain controls food intake and utilizes the potential energy stored in a “push and pull” manner [27]. The gastrointestinal microbiome can then influence the host through neural mechanisms. This is of particular importance when analyzing vagus nerve function in regulating eating behavior and weight, as well as the enteric nerve response to certain bacteria or bacterial metabolites. This association is noted in the inhibition of signals to the vagus nerve, causing significant weight loss. In contrast, vagus nerve activity appears to drive excessive eating behavior in murine experiments [28].

There are specific alterations in the gastrointestinal tract microbiome of obese phenotypes compared to lean phenotypes. The obese phenotype has abundant *Firmicutes *and fewer *Bacteroidetes *than lean individuals [29]. The obese phenotype patients also share a specific microbiome pattern, which is not fully identified. It appears to be transmissible, as demonstrated by the twin study of Turnbaugh [3].

The complexity of obesity and gastrointestinal tract microbiome is further demonstrated in the study by Jumpertz et al., stating that alteration of nutrient load can induce rapid microbiota changes, including an increase in *Firmicutes *and a decrease in *Bacteroidetes *[30].

Further research to strengthen the relationship of causality between the microbiota changes and obesity is an essential step in understanding the complexity of obesity.

Bariatric Surgery

**Figure 1:**
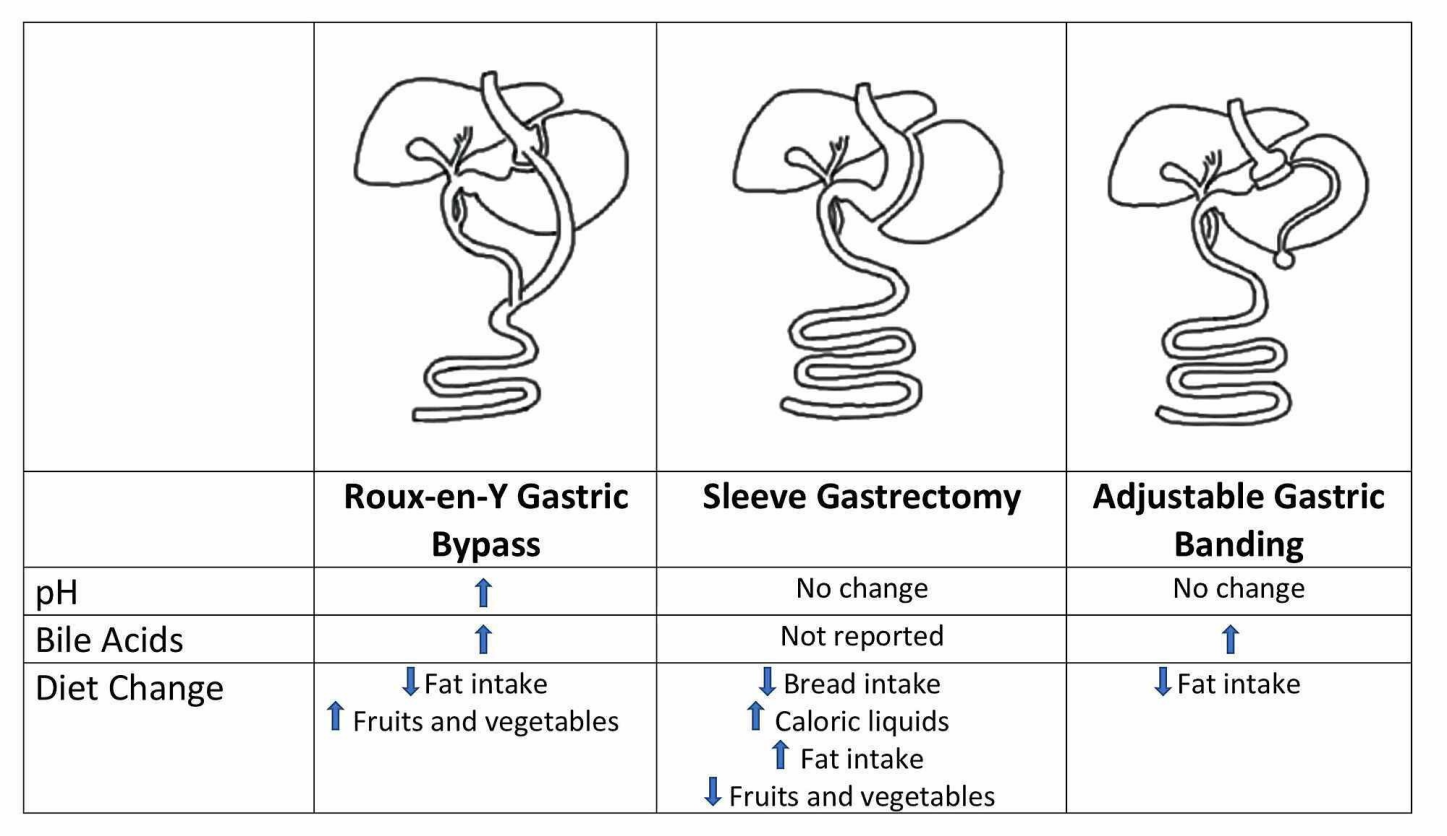
Comparison of outcomes in Roux-en-Y gastric bypass, sleeve gastrectomy, and adjustable gastric banding.

Bariatric surgery has always been known as the operation that creates anatomic changes to the gastrointestinal tract, aiding in weight loss and potential health-related gains [13]. It is currently the most effective treatment option for sustained weight loss and resolution of obesity-related comorbidities in individuals classified as BMI of ≥ 40 kg/m^2^ or ≥35 kg/m^2^ with obesity-related comorbidities [31]. There are numerous bariatric procedures, but this study only focuses on RYGB, SG, and AGB. Figure 1 lists some of the differences in the outcome between the three types of bariatric surgery.

RYGB rapidly rose in popularity and became the dominant bariatric procedure worldwide [13]. It provides both a restrictive and malabsorptive technique; it first involves creating a small pouch in the upper stomach that reduces the amount of food intake. Then the bowel is transected distal to the ligament of Treitz, creating the alimentary Roux limb, which is attached directly to the newly formed pouch. The food bypasses the distal stomach, duodenum, and proximal jejunum, which reduces the number of calories, fat, vitamins, and minerals absorbed.

Another commonly performed bariatric procedure is the SG, where the stomach is reduced by removing the stomach along the greater curvature, leaving the pylorus and creating a narrow gastric reservoir called the sleeve. It can induce significant weight loss by decreasing gastric volume, promoting early satiety, and restricting distention [32].

The last surgical technique to be discussed in this study is AGB. This involves placing an elastic silicone band around the top part of the stomach creating a small pouch that can be gradually adjusted. Adjustment of the band can be made by filling the balloon around the band with sterile saline. Eventually, the gap between the upper stomach space and lower stomach decreases, creating a restrictive effect and reducing nutrient uptake [33].

Possible Factors Affecting the Change in Microbiota After Bariatric Surgery

Many factors can influence microbiota change after bariatric surgery; the most commonly considered mechanism for the shift in dietary intake changes. Steinert et al. believed diet may have a strong influence on gut fungal composition [14]. The gut mycobiome, consisting predominantly of *Saccharomyces *and *Debaryomyces*, was found in study participants and is commonly found in mushrooms, cheese, bread, and beer. These findings were also supported by David et al. [33], where participants underwent a controlled diet study. The fungal composition was affected by food colonization of the same fungi species seen in participant fecal samples and cheese fed to the participants.

Remarkably, there is an association between alterations in the gut microbiome with appetite modifications, hedonic eating, and food preference. Sanmiguel et al. [25] utilized the Yale Food Addiction Scale (YFAS) for symptom count score and assessment of the desire to eat high- and low-calorie foods. Baseline YFAS scores showed they desired to eat high-calorie foods and undesired to eat low-calorie foods. After bariatric surgery, the patient’s desire to eat high- or low-calorie food was attenuated. There was a significant decrease in the YFAS that was directly associated with changes in the microbiota. Additional findings showed that there was also a decrease in the palatability of highly hedonic food. Palleja et al. [21] claimed a shift in food preference from high-calorie-dense foods toward low-calorie-dense foods after RYGB surgery.

Another factor reported to be affecting the microbiota is pH changes. There is a decrease in acid secretions after surgery, thus an increase in pH, due to the reduced size of the stomach, which makes the gastric barrier a better environment for the proliferation of oral microbiota [21]. In the study by Ilhan et al. [20], there were more facultative anaerobes and fewer anaerobes in the RYGB group, supporting that RYGB surgery increases the oxygen content of the stomach. There was also an increased abundance of oral cavity-associated microbes, suggesting that the microbes can bypass the stomach and its harsh environment and grow in the distal bowel.

Bile acids have also been considered a factor affecting the changes in microbiota after bariatric surgery. An anatomical factor that explains the increased bile acids seen in post-RYGB patients is attributed to the shortening of the route of enterohepatic circulation, which corresponds to the length of the biliopancreatic limb and the common limb [22]. Bile acids can determine the composition of the gut microbiome through the disruption of bacterial membrane integrity. Differences in surgical technique can lead to different levels of bile acid, as seen when comparing RYGB with AGB surgery. There is an altered bile flow after RYGB due to the anatomical rearrangement. RYGB creates an environment that stimulates the growth of bile-acid-transforming bacteria. On the other hand, AGB does not cause anatomical rearrangement, with no probable effect on bile acids [20]. Gutiérrez-Repiso et al. [8] observed that their success group patients, who maintained weight loss, had a bile-tolerant gut microbiome. While the weight loss failure group, who either regained weight or just had minimal weight loss, had a less bile-tolerant gut microbiome. Their study concluded that bile acid change led to the differences in cholesterol metabolism seen between the success and failure groups. The researchers also noted that the successful patients had increased bile salt hydrolase (BSH) activity compared to failure patients. An increase in BSH leads to an increase in the diversity of the systemic bile acids. Among the microbiota, the increase in *Firmicutes *phylum correlated with the BSH gene. The increase in BSH suggests that the microbiome can alter the levels of bile acid, which indicates that the relation of bile acids and the gastrointestinal microbiome is a two-way dynamic relationship that can affect one other [8].

Temporal Relation of Gut Microbiome and Bariatric Surgery

Current studies use fecal microbiota as evaluating changes in upper parts of the intestine poses an everyday obstacle. However, fecal microbiota represents the large intestine more, and dietary, medication, and type of surgical procedure can influence the sample [26]. This study notes the significant differences in gastrointestinal microbiome change represented by the fecal microbiome at different time points of the included studies.

For the first three months post-operatively, fecal microbiota mirrored the anatomic changes. The typical increase in proteobacteria, which plays a role in inflammatory processes, might be a marker of increased oxygen in the large intestine after the surgery [14]. In the same study, the inflammatory marker C-reactive protein was not elevated even though there was an increase in proteobacteria; thus, this increase is regarded as not significant. However, Al Assal et al. noted that the rise in proteobacteria was associated with higher insulin resistance and fat-rich diet intake [16]. It is important to note that the researchers could not find a relationship between proteobacteria and BMI, body weight, composition, or biochemical profiles.

Gastrointestinal microbiota plays a role in the changes in food preference seen in humans after bariatric surgery. According to Sanmiguel et al. [25], pleasure-driven eating decreased after bariatric surgery, which coincided with alterations in the gastrointestinal microbiome. This pleasure-driven eating is composed of high-calorie foods at baseline. The post-operative drop in food addiction scores was directly associated with *Catenibacterium *and *Anaerostipes *(phylum *Firmicutes*) and inversely correlated with *Butyricimonas*, *Enterococcus*, and *Odoribacter*. Studies note a decrease in *Firmicutes *and *Actinobacteria* at three months, but this is likely seen as a result of the anatomical change post-surgery that leads to a reduction in gastric acid secretion and in total energy intake with modification of nutrient composition [14]. Until six months, there was a decrease in *Firmicutes *and *Fusobacteria*, which are associated with pathogenic roles in esophageal cancer and inflammatory bowel disease. These were also found to be increased in obese gut microbiome pre-surgery [17]. There was no significant change between three months and one year. Providing further evidence that the gut microbiome shifting occurred mainly within the first three months and adapted to maintain levels up to one-year post-operatively [21].

The shift towards a healthier metabolism occurred within the first three months after RYGB and was maintained during the following nine months. However, the rate of improvement was markedly lower. Microbial diversity mirrored the change in metabolism [21]. Reduced microbial gene richness was noted to be prevalent in obese individuals compared to lean individuals. Thus, decreased diversity appears to be a marker of disease severity, as implicated in inflammatory intestinal diseases. Significantly increased microbial diversity was seen post-operatively immediately but regressed towards one year. This phenotype remained similar even after five years [19]. At one year post-operatively, there was a regression towards the pre-surgical levels. The regression toward pre-surgical groups was noted to be due to the adaptation of the microbiome. Although the gastrointestinal microbiome had regression towards pre-surgical levels, the whole body metabolism remained significantly improved or normalized [17,21].

Limited studies were conducted for one year from the procedure. Ilhan et al. [20] noted that at 2.9 ± 0.8 years, *Gammaproteobacteria*, *Bacilli*, and *Flavobacteria *were increased in relative abundance post-RYGB. *Gammaproteobacteria *and *Lactobacillus *have been associated with weight loss; however, *Fusobacteria *was not evaluated in this study in the context of weight loss. It is noted that *Flavobacteria *is less in the diabetic population than in non-diabetics.

Post-RYGB compared to post-AGB and pre-bariatric surgery had the highest microbial diversity among the three while pre-bariatric surgery had the lowest. The increased microbial diversity is attributed to structural changes that lead to the growth of facultative anaerobes [20]. *Streptococcus *was higher in successful weight loss individuals, which supports the hypothesis that increased gastric pH plays a role in shifting the gastrointestinal microbiota structure towards the oral microbiota [18].

Gutiérrez-Repiso et al. [8] concluded that individuals with successful weight loss had high microbiota diversity. They were monitored 8.3 ± 1.7 years from surgery, suggesting that microbial diversity further adapted to the new environment. In their study, out of the 24 patients who had undergone RYGB, only six patients had excess weight loss (EWL) 50% at nadir weight and throughout the follow-up period. While the remaining 18 patients either had EWL <50% or had weight regain. The successful EWL patients had a different core microbiota that can survive and adapt better to the new environment after bariatric surgery. The inability to adjust to the new environment can lead to dysbiosis. A particular change seen on EWL failure patients that were not noted on EWL success patients was the composition of EWL failure patients’ core microbiome, which comprised *Acinetobacter* and *Serratia* bacteria. These bacteria use carnitine as a mechanism of bile tolerance. Metabolites of carnitine are associated with increased atherosclerosis risk. It is also associated with dysregulation of the conversion of cholesterol into bile. This study suggests one out of numerous internal and external factors that may explain why some patients experience less weight loss or weight regain after the initial weight loss within one year of surgery. In a study by King et al. [34], among 1,406 who underwent bariatric surgery, the median rate of weight regain was 9.5% of the maximum weight lost one year after reaching nadir weight. Three years after reaching the nadir weight, the median weight regain was 22.5% and 26.8% five years after reaching nadir weight. This increase in weight regain suggests that adaptation to the new environment is a continuous process with tremendous progress for first year post-operatively. However, as you move further away from bariatric surgery, there is a decrease in the metabolic and gastrointestinal changes brought about by bariatric surgery due to external and internal factors discussed earlier. With this in mind, we can view bariatric surgery as a reset point to shift the gastrointestinal microbiota. A deeper understanding of the gastrointestinal microbiome changes and the factors that affect them may help the medical team provide patients better long-term care in treating the complex pathology of obesity.

Limitations

This study encountered difficulties in population size as most studies have a limited number of participants, which may have influenced the overall results. Larger cohorts studied for more prolonged periods might provide a better description of gut microbiota impacting health after bariatric surgery. This study did not account for the bias in gender, diet, and surgical technique. There was no specific diet among the studies. Most studies on bariatric surgeries did not assess the food intake before and after surgery. Future studies can explore the option of limiting bias brought about by food intake and digestion. The shift in gut microbiota after bariatric surgery is not one-dimensional. There is an interkingdom relationship between bacteria and fungi. This study did not take into account the fungal composition of the gastrointestinal tract. More work is needed in this field of study to deepen the understanding of the changes in the gut microbiome after bariatric surgery.

## Conclusions

Bariatric surgery is a known treatment for a specific set of type 2 diabetes mellitus and obese patients. The anatomic aspect of bariatric surgery as treatment is established and clearly stated; however, the dynamic component is yet to be defined entirely. This component includes alterations in the gastrointestinal microbiome, pH, bile, and dietary preferences, among other findings. This review indicates that the anatomic changes led to dynamic changes by comparing the gastrointestinal microbiome changes. There is less microbiome diversity seen in obese patients than the more diverse microbiome set seen in successful weight loss patients. Most of the research suggests that within three months after bariatric surgery, there is an increase in the *Proteobacteria *and *Bacteroidetes *phyla, along with a reduction in *Firmicutes*. These changes are reflective of the better metabolic levels seen in one year post-bariatric surgery patients. However, data that were taken further from the time of surgery have shown a regression trend leading to pre-surgical states in some patients. These patients ended up with less weight loss or some even had weight regain. This study demonstrated that we need more longitudinal observation to get a deeper understanding of the pathology of obesity and reaction to bariatric surgery to better take care of our patients by providing continuation of care catered to the new environment brought about by bariatric surgery.
